# Return to work 10 years after severe trauma

**DOI:** 10.1007/s00068-025-02950-3

**Published:** 2025-08-22

**Authors:** Annemarie Rusche, Adelina Denzel, Annette Keß, Nikolas Schopow, Christian Kleber, Georg Osterhoff

**Affiliations:** Department of Orthopaedics, Trauma and Plastic Surgery, University Hospital Leipzig, Liebigstrasse 20, 04103 Leipzig, Germany

**Keywords:** Polytrauma, Return to work, Mental health, Rehabilitation, Quality of life

## Abstract

**Background:**

Severe trauma continues to pose a substantial burden on survivors, particularly in terms of long-term physical, psychological, and social functioning. While survival rates have improved, data on long-term outcomes remain limited. This study evaluates ten-year post-injury outcomes in patients with major trauma, focusing on return to work and social participation.

**Methods:**

In this single-center, retrospective cohort study, adult patients (≥ 18 years) with an Injury Severity Score (ISS) ≥ 9 treated between 2010 and 2013 were surveyed and distributed minimally 10 years later. Patients completed standardized questionnaires assessing sociodemographic and occupational data, functional status, and psychological well-being using the Trauma Outcome Profile (TOP).

**Results:**

Ninety-one patients completed the follow-up. The mean age at injury was 43.0 years, with a mean ISS of 20.8. Ten years post-trauma, 82.4% of patients had returned to work; 10.6% required vocational retraining, and 25.3% changed occupations. Failure to return to work was significantly associated with higher ISS (*p* = 0.027), increased anxiety (*p* = 0.005), post-traumatic stress disorder (PTSD, *p* = 0.039), and reduced mental functioning (*p* = 0.009), but not with physical functioning ten years after the trauma. Patients with mental health impairments were more likely to experience reduced independence, impaired social participation, and difficulties in activities of daily living.

**Conclusion:**

A majority of patients successfully reintegrated into the workforce ten years after trauma. Mental health, rather than physical disability, emerged as the primary determinant of long-term occupational reintegration. These findings underscore the necessity for comprehensive, long-term rehabilitation programs that prioritize psychosocial support.

## Introduction

Severe trauma remains a major public health issue, significantly impacting survivors’ activities of daily living and their ability to return to work. Although advancements in prehospital and in-hospital trauma care over recent decades have markedly improved survival rates research on the long-term consequences faced by these patients after trauma is lacking [1]. A trauma not only affects physical health but also has profound implications for psychological well-being and social reintegration [2]. Survivors often struggle with lower physical and mental health, which limits their full participation in society. Previous studies have shown that polytrauma is linked to increased long-term disability and heightened psychological distress [3]. In the context of polytrauma – defined as injuries affecting two or more organ systems, with at least one injury or their combination being life-threatening – these challenges are amplified. Such patients often require prolonged rehabilitation involving complex, multidisciplinary approaches tailored to individual needs [2].

Despite existing literature exploring short- and medium-term outcomes, there remains a paucity of research examining recovery beyond five years, particularly regarding factors that influence return to work [3, 4].Questions persist about the characteristics that enable patients to successfully reintegrate into the workplace, as well as the risk factors with failure to do so.

This study aims to analyze ten year follow-up data of individuals who have endured trauma, focusing on key aspects such as workforce reintegration, career changes, and factors that hinder return to work. The study seeks to provide valuable insights into the clinical management and rehabilitation planning of trauma patients.

## Materials and methods

### Patients

This single-center retrospective study included all adult patients with Injury Severity Score (ISS) ≥ 9 and age ≥ 18 years who were admitted to the trauma room of a level 1 trauma center between 01/2010 and 12/2013. Exclusion criteria were the absence of a permanent residence permit in Germany and objecting to the use of personal data for research purposes.

### Data acquisition

A total of 503 patients were contacted by mail and, after providing informed consent, asked to complete self-administered questionnaires collecting data on epidemiological parameters, data on education and socioeconomic status. These questionnaires were distributed minimally after ten years and contained information on return to work after trauma, the need for vocational retraining and career changes. The type of work and level of education prior to the trauma were also assessed, as well as the Trauma Outcome Profile (TOP). The TOP is an instrument specifically designed to assess health-related quality of life after trauma. It comprises ten subscales addressing the following dimensions: depression, anxiety, PTSD, social interaction, pain before and after trauma (in 14 body regions, rated on a 0–10 scale), physical functioning before and after trauma (in 14 body regions, rated on a 0–10 scale), daily activities, mental functioning, body image and overall life satisfaction.

### Statistical analysis

All data were recorded in an Excel database (Microsoft Corp., Redmond, WA, USA) and exported to SPSS 29.0 (IBM Corp., Armonk, NY, USA) for statistical analysis. Effect sizes were calculated where applicable, with Cohen’s *d* interpreted as follows: small effect (≥ 0.2), medium effect (≥ 0.5), and large effect (≥ 0.8).

Demographic and clinical data are presented as means with standard deviation (SD) for continuous variables and as absolute frequencies (n) and percentages for categorical variables. Shapiro-Wilks test was performed to assess normality. In cases of skewed distributions, the median and interquartile range (IQR) are also provided. Group differences in continuous variables were assessed using Student’s *t*-test or the Mann-Whitney *U* test, as appropriate. Categorical variables were analyzed using the chi-squared test. A *p-value* < 0.05 was considered statistically significant.

## Results

### Demographics

**Fig. 1:**
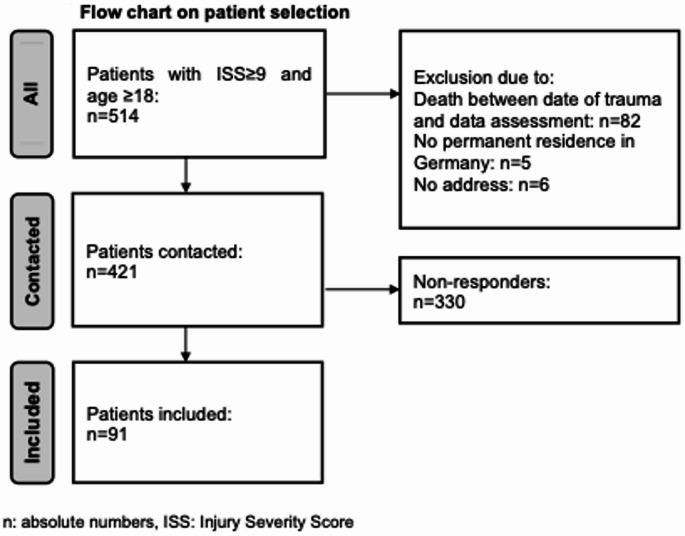
Flow chart on patient selection

A total of 91 patients with completed questionnaires and available clinical data were included in the analysis. Further details on patient selection are displayed in the flow chart below (Fig. 1). The mean age at the time of initial trauma was 43.0 years (SD 17.2), and 76.9% of the cohort were male. The mean ISS was 20.8 (SD 12.4). The average hospital length of stay was 17.1 days (SD 13.8). In total, 81 patients (89.0%) required intensive care treatment. The mean length of ICU stay was 7.0 days (SD 10.3). Respiratory support via intubation and mechanical ventilation was necessary in 39 patients (42.9%). Among these, the mean duration of mechanical ventilation was 2.6 days (SD 5.6). Long-term functional outcomes were assessed using the Glasgow Outcome Scale (GOS): severe disability (GOS 3) was observed in six patients (6.5%), moderate disability (GOS 4) in 26 patients (28.6%), and good recovery (GOS 5) in 59 patients (64.8%).

### Pattern of injuries

The most frequently affected anatomical region was the extremities and pelvic girdle, involved in 72.5% of cases. Thoracic injuries were the second most common, affecting 62.6% of severely injured patients. Head and neck trauma was documented in 49.5% of cases, while abdomen and pelvic organ injuries were observed in 41.8%. Facial injuries and external trauma ranked fifth and sixth, affecting 20.8% and 15.4% of patients.

### Pain and functional deficit

More than half of the patients (51.6%) reported significant pain ten years after trauma, rating it above 5 on a 0–10 scale. Severe functional impairments were documented in 43 patients (47.2%).

The extremities, pelvic girdle, and spine were the most affected areas for both persistent pain and functional limitations. Significant pain reported 41 patients (45.1%) in the extremities and pelvic girdle, while 26 patients (28.6%) reported spinal pain.

Functional deficits followed a similar pattern, with 38 patients (41.7%) experiencing deficits in the extremities and pelvic girdle, and 36 patients (39.6%) reporting functional limitations in the spine. Pain and functional impairment in the chest and abdomen were reported only by a minority of patients (Table 1).

**Table 1:** Pain and functional deficit ≥ 5 on a self-rating scale (0–10) ten years after trauma

Affected anatomic region	Pain	Functional deficit
Body	47 (51.6%)	43 (47.2%)
Head/Neck	18 (19.7%)	10 (11.0%)
Chest	5 (5.5%)	3 (3.2%)
Abdomen	3 (3.2%)	2 (2.2%)
Extremities/pelvic girdle	41 (45.1%)	38 (41.7%)
Spine	26 (28.6%)	36 (39.6%)

Pain and functional deficit on a self rating scale (0–10), ranking from 0 (no problems) to 10 (severe problems)

### Trauma outcome profile

The mean scores indicate the severity of symptoms or impairment in different psychological, social, and physical health domains. Patient satisfaction ranked highest with mean score of 88.2 (SD 20.6). This was followed by daily activities and body image, with a mean scores 83.0 (SD 24.4) and 82.1 (SD 32.0), respectively. Mean Depression was 77.8 (SD 27.2), anxiousness 78.6 (SD 28.3), and post-traumatic stress disorder (PTSD) 76.9 (SD 26.1), showing similar values. Mental functioning had the lowest mean, at 62.0 (SD 32.2).

### Return to work

Seventy-five patients (82.4%) returned to work. Of those, eight patients (10.6%) underwent vocational retraining within their original occupation, and 19 patients (25.3%) changed careers as a consequence of their injuries. A total of 16 patients (17.6%) did not resume employment following severe trauma. Patients who did not return to work had a higher initial ISS compared to those who did not (26.1, SD 16.6 vs. 19.4, SD 11.1, *p* = 0.027). However, both groups demonstrated similarly good physical function ten years after trauma (Table 2).

Patients with anxiety (*n* = 28) were significantly less likely to return to work after experiencing severe trauma compared to those without anxiety (*p* = 0.005, OR 5.0, 95% CI: 1.6–15.7). Notably, 35.7% of individuals with severe anxiety did not return to work, whereas only 10.0% of those without anxiety faced the same outcome. Patients experiencing impaired mental function (*n* = 52) demonstrated a significantly lower likelihood of returning to work compared to those with unimpaired mental function (*p* = 0.009, OR 6.3, 95% CI: 1.3–29.6). Specifically, 26.9% of patients with impaired mental function did not resume employment, in contrast to 5.6% of those without mental impairments. Patients diagnosed with post-traumatic stress disorder (PTSD) (*n* = 35) exhibited a significantly higher likelihood of remaining unemployed compared to those without PTSD (28.6% vs. 11.3%, *p* = 0.039, OR 3.1, 95% CI: 1.0–9.6). Pain, sex and age were not different among the two groups (Table 2). The use of walking aids had no significant effect on the return to work (*p* = 0.161).

**Table 2:** Description of the population who did not return to work

Characteristic	No RTW	RTW	*p*
Age	37.6 a (SD a 17.3)	44.1 a (SD a 17.1)	0.190
Sex (female)	3 (18.8%)	16 (22.2%)	1.0
ISS	26.1 (SD 16.6)	19.4 (SD 11.1)	0.027
Pain ^1^	1.7 (SD 1.0)	1.5 (SD 0.9)	0.304
Functional deficit ^2^	1.8 (SD 1.0)	1.6 (SD 0.9)	0.465
Anxiety ^3^	10 (62.5%)	18 (25.0%)	0.005
Impaired mental function ^4^	14 (87.5%)	38 (85.8%)	0.009
PTSD ^5^	10 (62.5%)	25 (37.7%)	0.039

RTW: Return to Work; y: years, ISS: Injury Severity Score

Continuous data is reported as means with standard deviation (SD), categorical data as absolute numbers (n) and percentages. ^1^Pain was measured using visual analogue scale, ranking from 0 (no pain) to 10 (extreme pain)

^2^functional deficit as reported by the patients on a scale from 0 (no function) to 10 (good function), ^3^TOP anxiety < 80, ^4^TOP mental function < 80, ^5^TOP PTSD < 80. TOP score ranges from 0 to 100, where higher scores indicate better health-related quality of life

### Vocational retraining and career change

Individuals with impaired mental function (*n* = 53) were more likely were to undergo vocational retraining compared to those with better mental function (15.1% vs. 0%, *p* = 0.013, OR 1.2, 95% CI 1.1–1.3). Similar results were observed in patients with PTSD (*n* = 36). Retraining was necessary in (19.4% vs. 1.8%, *p* = 0.007, OR 12.6, 95% CI 1.5-107.1). Neither self-employment (*p* = 0.331) nor completion of an academic education (*p* = 0.134) had a significant effect on the likelihood of vocational retraining post trauma. But patients who had completed an academic education prior to the trauma (*n* = 18) were significantly less likely to change their career compared to patients without an academic background (5.6% vs. 27.7%, *p* = 0.040).

### Social interaction and participation

Several risk factors contributing to impaired social interaction were identified. Anxiety (*p* < 0.001, OR 11.0, 95% CI 3.9, 31.1), depression (*p* < 0.001, OR 5.8, 95% CI 2.2–14.9) and PTSD (*p* < 0.001, OR 9.2, 95% CI 3.5–24.3) significantly influenced social participation. Patients who were unable to work exhibited a higher likelihood of experiencing impaired social interaction (*p* = 0.003, OR 6.0, 95% CI 1.7–20.6). Similar associations were observed among individuals requiring vocational retraining (*p* = 0.007, OR 12.5, 95% CI 1.5-107.1) or a career change (*p* = 0.013, OR 3.8, 95% CI 1.3-11.05). Financial problems were associated with reduced social engagement (*p* = 0.001, OR 4.6, 95% CI 1.8–11.9). In contrast, completion of an academic education was found to be a protective factor for maintaining social engagement following severe trauma (*p* = 0.011, OR 3.7, 95% CI 0.2–0.9).

### Activities of daily living

Paraplegia (*p* = 0.019, OR 12.4, 95% CI 1.3–117.0) and impaired physical function (*p* < 0.001, OR 5.2, 95% CI 1.9–14.2) were significantly associated with reduced ability to perform ADL. All patients requiring walking aids were more likely to have impaired ADL (*p* < 0.001, OR not applicable). Individuals experiencing pain (*p* = 0.001, OR 6.6, 95% CI 1.3–9.3) and anxiety (*p* < 0.001, OR 5.1, 95% CI 1.9–13.7) had a higher likelihood of ADL impairment. Financial difficulties also adversely affected the ability to manage ADL (*p* = 0.027, OR 2.9, 95% CI 1.1–7.9).

### Independency

Impaired independence was more prevalent among patients with paraplegia following severe trauma (*p* = 0.011, OR 15.1, 95% CI 1.6-143.7). The use of walking aids (*p* < 0.001, OR 18.5, 95% CI 5.3–64.2) was identified as a significant factor associated with reduced autonomy. Anxiety (*p* < 0.001, OR 7.7, 95% CI 2.7–22.4) and PTSD (*p* = 0.002, OR 4.6, 95% CI 1.6–12.8) were likewise linked to impaired independence.

## Discussion

This study investigated the long-term outcomes of patients who have experienced severe trauma, focusing on their reintegration into the workforce, potential career changes, and potential risk factors resulting in inability to work. Key findings include the importance of good mental health on the ability to return to work, as opposed to physical impairment.

In this study, 82% of patients were able to return to work after trauma, although patients had relatively high mean ISS, indicating severe traumatic injury. This percentage is similar to that from international literature, reporting 56.5 to 79.3% of patients returning to work [5]. Physical impairments, such as paraplegia and the necessity of walking aids, negatively impacted patients’ independence. However, these factors did not influence the likelihood of returning to work in this study, possibly due to the limited number of patients with paraplegia.

Patients showed relevant mental impairment in all domains of TOP. In this study, 62.5% of patients who did not return to work had PTSD. A study by Yue et al. reported that 42% of patients reported positive for PTSD, major depression or both six months after polytrauma [3]. Despite the high prevalence of PTSD, effective screening tools and accessible prevention programs remain limited [6, 7]. A recent systematic review showed that targeted psychological interventions have a positive short-term impact on PTSD, depression and anxiety following traumatic injury, underlining the need for further research in this field [8].

Although women are two to three times more likely to develop PTSD after major trauma, gender did not influence the likelihood returning to work following sever trauma, which is consistent with previous literature [9].PTSD is a potential risk factor for remaining unemployed, however women tend to return to work more [10]. The discrepancy observed between higher PTSD rates in women and comparable return to work outcomes may be due to the increased use of psychosocial support after trauma [9]. Another possible explanation is the nature of patients’ pre-trauma job; however, this aspect could not be assessed due to a lack of detailed occupational data.

While the influence of pre-trauma occupation could not be evaluated due to limited data, the broader socioeconomic impact of severe trauma remains evident. Previous literature showed that costs for acute polytrauma care are high [11]. Long-term expenditures must also be considered. Post-hospital care, rehabilitation, and productivity loss can account for up to 50% of the total cost of care [12]. Especially post-acute care, involving reintegration into society and the working market often require coordinated, multidisciplinary rehabilitation efforts [13]. Although this need is well recognized, evidence-based data remains limited [14].

In the context of major trauma, patients often face severe injury with potential physical impairment and psychological conditions, such as PTSD and anxiety, all of which can lead to longer recovery and higher hospital costs. Understanding the determinants that influence long-term outcome after major trauma is crucial for recovery and social reintegration. By examining these factors, the findings of this study provide valuable insights that may inform and optimize rehabilitation strategies, improving both mental and physical health. Ultimately, this can facilitate better social reintegration after severe trauma and support a return to the labor market. Further research is needed to explore targeted strategies for individuals at risk of not returning to work, with the goal of enhancing their chances of labor market and social participation.

This study has some limitations. First, only 91 patients from a single center were involved in this study, representing only a small subset of all patients with traumatic injury during the study period. As participation was voluntary, response bias cannot be ruled out, potentially favoring individuals with particularly positive or negative experiences. The broad confidence intervals, most likely due to the small and heterogeneous cohort, highlight the need to cautious data interpretation. Due to the nature of a retrospective study, the need for intensified rehabilitation could not be prospectively assessed. Moreover, no standardized screening was performed to identify patients at risk for post-traumatic mental impairment, which may have influenced RTW outcomes.

## Conclusion

Most patients successfully returned to work ten years after trauma. Mental health, rather than physical impairment, emerged as the key determinant of long-term occupational reintegration. Anxiety, PTSD, and impaired mental functioning were strongly associated with failure to return to work, reduced independence, and limited social participation. These findings highlight the need for structured, long-term rehabilitation programs with a strong psychosocial focus. Early identification of at-risk individuals may improve long-term outcomes and support sustainable reintegration into work and society.

## Data Availability

No datasets were generated or analysed during the current study.
